# Preparation and Characterization of Thin-Film Composite Membrane with Nanowire-Modified Support for Forward Osmosis Process

**DOI:** 10.3390/membranes5010136

**Published:** 2015-03-20

**Authors:** Ze-Xian Low, Qi Liu, Ezzatollah Shamsaei, Xiwang Zhang, Huanting Wang

**Affiliations:** Department of Chemical Engineering, Monash University, Clayton VIC 3800, Australia

**Keywords:** forward osmosis (FO), polyamide, polyethersulfone (PES), thin film composite (TFC), internal concentration polarization (ICP), membrane, germanate

## Abstract

Internal concentration polarization (ICP) in forward osmosis (FO) process is a characteristic problem for asymmetric thin-film composite (TFC) FO membrane which leads to lower water flux. To mitigate the ICP effect, modification of the substrates’ properties has been one of the most effective methods. A new polyethersulfone-based ultrafiltration membrane with increased surface porosity and high water flux was recently produced by incorporating Zn_2_GeO_4_ nanowires. The composite membrane was used as a substrate for the fabrication of TFC FO membrane, by coating a thin layer of polyamide on top of the substrate. The substrate and the nanowires were characterized by a range of techniques such as SEM, XRD, and contact angle goniometry. The water permeability and molecular weight cut-offs (MWCO) of the substrate; and the FO performance of the TFC membrane were also determined. The Zn_2_GeO_4_-modified membrane showed ~45% increase in water permeability and NaCl salt rejection of 80% under RO mode. In FO mode, the ratio of water flux to reverse solute flux was also improved. However, lower FO flux was obtained which could be due to ICP. The result shows that Zn_2_GO_4_ nanowire may be used as a modifier to the substrate to improve the quality of the polyamide layer on the substrate to improve the flux and selectivity, but not as effective in reducing ICP. This work demonstrates that the incorporation of nanomaterials to the membrane substrate may be an alternative approach to improve the formation of polyamide skin layer to achieve better FO performance.

## 1. Introduction

Global water crisis and clean water shortage have remained one of many pressing development challenges of all time. Several measures to alleviate the stresses on water supply should be implemented such as water conservation, improved catchment and distribution systems but they could only improve the use of existing water resources, not increase them [1]. The only ways to increase water supply beyond that which is available from the hydrological cycle are desalination and water reuse [2]. Forward osmosis (FO) desalination, one of the current emerging technologies is able to utilize alternative source of energy (such as solar and waste heat) and is less prone to membrane fouling compared to pressure-driven membrane separation process such as reverse osmosis (RO). FO desalination utilizes osmotic pressure difference between a feed solution (FS) and a draw solution (DS) to drive water across a semi-permeable membrane [3]. Besides desalination, other potential applications for FO technology have also been reported including wastewater treatment [4], biomass concentration [5], and pharmaceutical applications [6].

Despite the high potential of FO technology, several technological barriers such as internal concentration polarization (ICP) and fouling have yet to be overcome [7]. ICP is a phenomenon inherent of the osmosis driven membrane system, and is caused by the impeded diffusion of solutes within the porous support layer of the membrane which reduce the overall driving force across a membrane [8]. ICP effect is observed, when thin film composite (TFC) membrane, which is designed for pressure-driven membrane processes, is used for FO application. The thick and dense support layers, necessary to withstand large hydraulic pressures causes ICP [9]. Because this ICP effect is unperturbed by stirring [10], modifying the support layers is essential to reduce ICP effect in TFC membrane [9]. Ideally, FO membrane should comprise the following characteristics: (1) an active layer with high water permeability and low solute permeability (high flux, high salt rejection), (2) a support layer with smaller structural parameter (highly porous, thin, chemically and mechanically stable) [11], and (3) a hydrophilic membrane substrate [12]. 

Many attempts have been made to reduce the ICP effects, including optimization of the structure of substrate (controlling the phase inversion process) [7,13,14,15,16], polymeric substrate modification (chemical modification [12,17] or incorporating nanomaterials [18,19,20,21]), alternative substrate fabrication method (electrospinning nanofibers substrate [22,23,24,25,26,27,28] or using double-blade casting technique [29]), and redesign of the FO membrane structure, such as a double-skinned FO membrane [30] or dual-layer membrane [31]. In this work, we use Zn_2_GeO_4_-nanowire-modified polyethersulfone (PES) ultrafiltration (UF) membrane as the substrate for FO membrane. Zn_2_GeO_4_-nanowire-modified PES membrane exhibits increased number of nanopores in the active layer leading to enhanced water flux [32], which may be beneficial as a substrate for FO membrane. The modified PES substrate may exhibit better FO performance either via (i) the reduction in ICP by increasing porosity or reducing thickness and tortuosity; or (ii) formation of a polyamide layer with higher quality. The FO results were compared to pristine PES membrane to determine the suitability of the modified substrate for FO application. The incorporation of nanomaterials to the membrane substrate may be an alternative approach to improve the formation of polyamide skin layer to achieve better FO performance

## 2. Results and Discussion

### 2.1. Characterization of Nanowire, Substrates and Membranes

**Figure 1:**
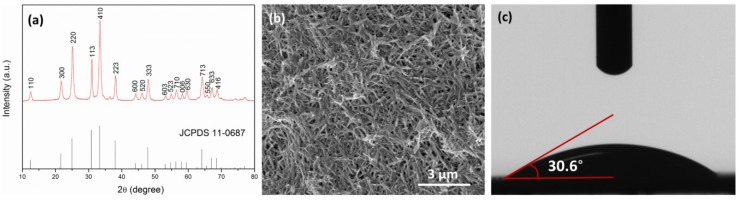
(**a**) XRD pattern of Zn_2_GeO_4_ nanowires; (**b**) SEM images of Zn_2_GeO_4_ nanowires; (**c**) contact angle measurement of Zn_2_GeO_4_ pellet.

The X-ray diffraction (XRD) patterns of the as-synthesized samples are shown in Figure 1a.The diffraction peaks in the XRD pattern of Zn_2_GeO_4_ nanowires can be assigned to the rhombohedral phase of Zn_2_GeO_4_ (JCPDS 11-0687) with lattice constants of *a* = *b* = 1.423 nm and *c* = 0.953 nm, *α* = *β* = 90° and *γ* = 120°. Figure 1b shows the low-magnification SEM images of the nanowires, where the individual nanowires have a length between 200 and 300 nm and uniform diameter of 20–50 nm. 

**Figure 2:**
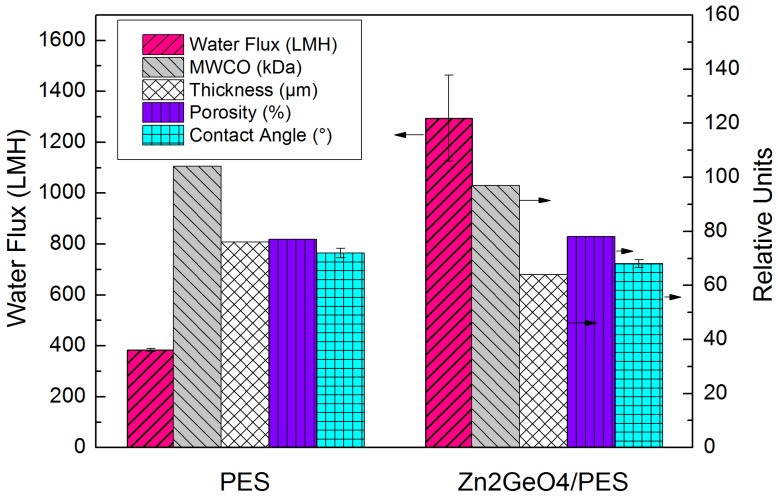
Ultrafiltration (UF) performance, thickness, porosity and contact angle of pristine polyethersulfone (PES) membrane and Zn_2_GeO_4_/PES membrane.

Figure 2 shows the characteristics of the pristine UF membrane (pure PES) and Zn_2_GeO_4_/PES membrane. The modified membrane showed ~3.5 times higher pure water flux than control membrane and also slightly improved molecular weight cut off (MWCO). The increase in water flux is due to the increased number of pores in the active layer [32]. The porosity of the modified membrane remained almost the same as the control membrane. After incorporating Zn_2_GeO_4_ nanowires, the membrane also becomes slightly more hydrophilic as shown by the reduced contact angle. The modified membrane also showed a slight decrease in membrane thickness, which may be due to changes in polymer dope solution. The modified membrane was used as the substrate for TFC FO membrane. A thin layer of polyamide was formed via interfacial polymerization on top of the modified PES substrate. 

### 2.2. FO Membrane Performance

**Figure 3:**
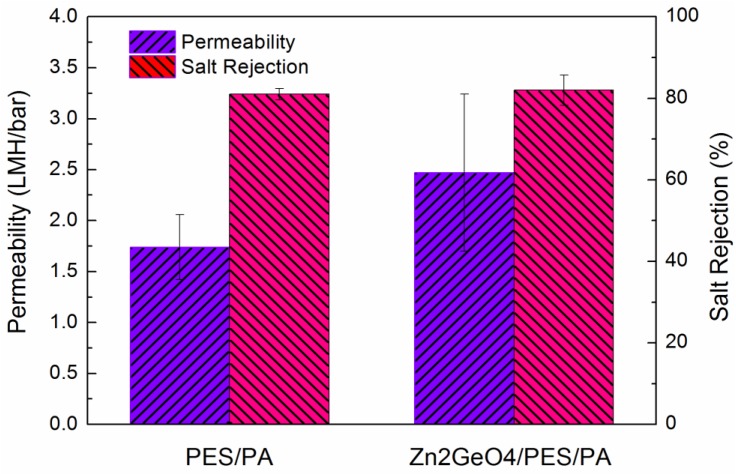
Water permeability and NaCl rejection of pristine membrane and Zn_2_GeO_4_/PES/PA forward osmosis (FO) membrane in reverse osmosis (RO) mode.

The effect of Zn_2_GeO_4_ on membrane water permeability and salt rejection were evaluated in RO mode. Figure 3 shows the RO performance of both pristine and modified the membrane. The modified membrane shows ~45% increase in pure water permeability and slight increase in the salt rejection. This indicates a higher quality of polyamide formed on top of the membrane which will be discussed with the FO result. The permeability and salt rejection of three commercial membranes along with those reported elsewhere (CTA-HW [11], CTA-W [7], and CTA-NW [7]) and the membranes from current work are shown in Table 1. The three membranes were all based on cellulose triacetate (CTA). From Table 1, TFC membrane performed better than CTA membrane in RO mode due to its thin active layer. However, the results from the commercial membranes should not be directly compared since the RO test was done in different conditions.

**Figure 4:**
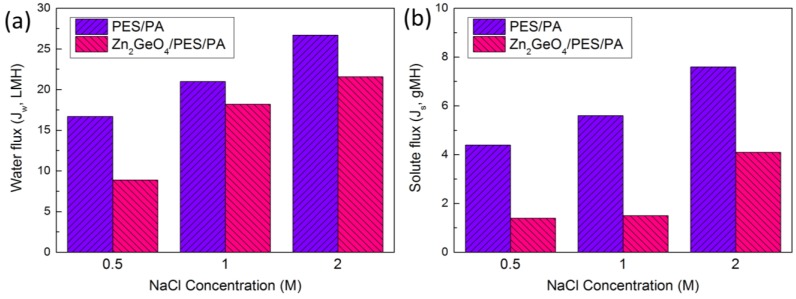
(**a**) FO water flux; (**b**) solute flux of pristine membrane and Zn_2_GeO_4_/PES/PA membrane using NaCl at different concentration (orientation: active-layer-facing-FS (AL-FS)).

**Figure 5:**
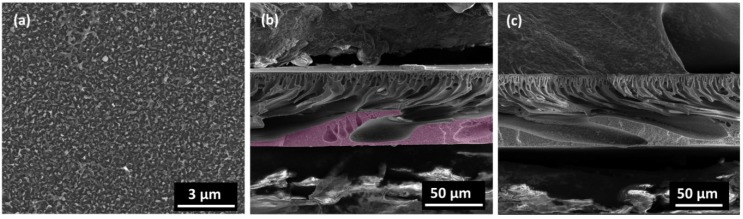
(**a**) SEM images of the TFC FO membrane active layer; (**b**) Zn_2_GeO_4_/PES; (**c**) PES membrane cross section. The highlighted section indicates the thick pore wall.

The FO performance was determined by using 0.5 M, 1.0 M and 2.0 M NaCl solution as the draw solution, and DDI water as the feed solution. In FO mode (Figure 4), the modified membrane shows lower water flux and lower reverse solute flux, *i.e.*, higher salt rejection, despite higher water permeability and salt rejection in RO mode. This indicates that incorporation of Zn_2_GeO_4_ nanowires on the membrane substrate does not effectively reduce the ICP as intended. On the contrary, the opposite result was obtained as shown in Figure 4a, where a non-linear relationship between water flux and NaCl concentration is obtained. This might be due to the thicker pore wall within the membrane matrix, which leads to higher tortuosity. Figure 5 shows the typical “ridge-and-valley” morphology of the polyamide layer and the cross-sectional view of the modified membrane. The increased ICP could be partly caused by the thick pore walls near the bottom matrix of the modified membrane (Figure 5b), which may also increase the tortuosity of the membrane. For the control membrane (Figure 5c), pore walls at the bottom matrix appear thinner and the widths of the finger-like pores are smaller. The results are also consistent with the structural parameter calculated from osmotic flux tests, where the S value of the modified membrane increased from 352 to 540 µm. The corresponding *τ* of the modified membrane is 6.58, as compared to 3.56 of the control membrane (Table 2). Despite lower FO flux, the overall ratio of the water flux to solute flux (J_w_/J_s_) is improved (Table 2). This might be due to the changes in the surface physical and chemical property which improve the interfacial polymerization of the polyamide layer leading to the formation of the polyamide layer with higher permeability and salt rejection. It is widely established that the properties of the support layer such as hydrophilicity has an influence on the properties of the polyamide layer and the overall separation performance [33,34,35,36]. The Zn_2_GeO_4_ nanowires showed a contact angle of 30.6° (average of three measurements), indicating hydrophilic surface of the nanowires (Figure 1c). The modified membranes which showed slight improvement over surface hydrophilicity and surface porosity may facilitate MPD monomers adsorption within the porous substrate. The monomers eventually diffuse out from the pores and react with acid chlorides (TMC). Higher adsorption of MPD monomers may produce a more compact polyamide layer. On the other hand, the hydrophilic Zn_2_GeO_4_ nanowires may interact with MPD monomers, which may reduce the diffusion rate of MPD monomer during the interfacial polymerization. Since interfacial polymerization between MPD and TMC occurs predominantly in the organic phase, the slow diffusion may improve the stability of the polyamide layer on the support layer [34]. The existence of the nanowires may also affect the degree of crosslinking of the polyamide layer. The changes in the substrate properties and chemical interactions by incorporating Zn_2_GeO_4_ nanowires may be the reason to the formation of the higher quality of polyamide layer which increases the J_w_/J_s_ ratio of the TFC FO membrane.

**Table 1:** Properties of control, modified and commercial membranes.

Sample	Water permeability (A; LMH/bar)	NaCl Rejection (%)	Salt permeability (B; LMH)	B/A (bar)	Ref.
PES^a^	1.74 ± 0.32	81.0	6.3	3.62	current work
Zn_2_GeO_4_/PES^a^	2.47 ± 0.77	82.0	8.4	3.40	current work
CTA-HW^b^	1.19 ± 0.19	78.5	0.09	0.08	[7]
CTA-W^b^	0.33 ± 0.04	81.9	0.01	0.03	[7]
CTA-NW^b^	0.46 ± 0.07	92.4	0.01	0.02	[7]

^a^ The water permeability was evaluated in RO mode at applied pressure of 15.5 bar (225 psi) with DDI water. The salt rejection was evaluated at 15.5 bar with 34 mM NaCl (2000 ppm) as feed solution; ^b^ The water permeability was evaluated in RO mode over an applied pressure ranging of 1–5 bar with ultrapure water. The salt rejection was evaluated at 3.75 bar with 20 mM NaCl as feed solution.

**Table 2:** Ratio of J_w_/J_s_ at different concentration of NaCl, *K*_m_, *S*, *τ* of control and modified membranes.

Sample/ DS Concentration (M)	0.5	1.0	2.0	*K*_m_ (10^5^ s/m)	*S* (µm)	*τ*
Control	3.8	3.8	3.5	2.2	352	3.56
Zn_2_GeO_4_/PES/PA	6.4	12.1	5.3	3.4	540	6.58

## 3. Experimental Section 

**Chemical.** Polyethersulfone (PES, Ultrason E6020P, 51 kDa) was purchased from BASF Co. Ltd., Germany. 1-methyl-2-pyrrolidinone (NMP; 99.0%), n-hexane (anhydrous, 95%), polyethylene glycol (PEG), germanium (IV) oxide (GeO_2_; ≥99.99%), zinc acetate dihydrate (of Zn(CH_3_COO)_2_·2H_2_O; reagent grade) and tetramethylammonium hydroxide (TMAOH; 25 wt.% in H_2_O), 1,3,5-benzenetricarbonyl trichloride (TMC; 98%), 1,3-phenylenediamine (MPD, ≥99%) were purchased from Sigma-Aldrich, Australia. Sodium chloride (NaCl) was purchased from Merck Millipore, Australia). The water used for the experiments was purified with a water purification system (Milli-Q integral water purification system, Merck Millipore Australia) with a resistivity of 18.2 MΩ/cm. Distilled water was obtained from a laboratory water distillation still (Labglass Aqua III).

**Synthesis of zinc germanate (Zn_2_GeO_4_) nanowire.** All the chemicals were used as received without further purification. In a typical synthesis of Zn_2_GeO_4_ nanowires, 0.52 g of GeO_2_ (2.5 mmol) and 1.10 g of Zn(CH_3_COO)_2_·2H_2_O (5 mmol) were added to a 25% tetramethylammonium hydroxide (TMAOH) aqueous solution (15 mL). The mixture was stirred for 40 min and then transferred to a Teflon-lined stainless steel autoclave of 25 mL inner volume. The hydrothermal synthesis was performed under an auto-generated pressure at 180 °C for 12 h in a convection oven, followed by natural cooling to room temperature. The product was collected by centrifugation, washed thoroughly with deionized water and alcohol for several times, and then dried at 60 °C for 12 h. A white Zn_2_GeO_4_ powder was obtained.

**Figure 6:**
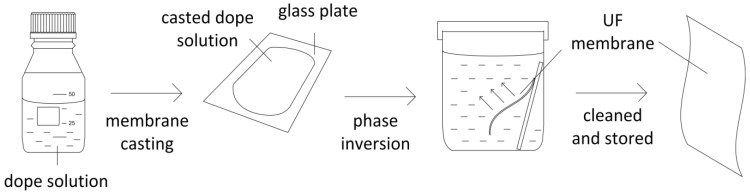
Schematic diagram of UF membrane fabrication via phase inversion.

**Preparation of membrane support.** The Zn_2_GeO_4_/PES composite membranes were prepared via non-solvent induced phase separation at room temperature. The casting solution was prepared by first mixing 0.018 g of Zn_2_GeO_4_ in 34 g of NMP. The mixture then underwent 90 min ultrasonication, followed by 30 min stirring to ensure good dispersion. Then, 6 g of PES was added, and the mixture was stirred until all PES was dissolved. The solution was left to degas overnight before use. The composition of the pristine and modified membrane substrate is shown in Table 3. The membranes were cast on a glass plate using an adjustable micrometer film applicator (stainless steel blade at a gap of 150 µm, Gardco, Pompano Beach, USA) at room temperature (Figure 6). The cast solution underwent air evaporation for 10 s before being immersed in a coagulation bath of distilled water overnight. The membranes were then removed from the bath, rinsed thoroughly with double-deionized (DDI) water and stored in fresh DDI water before use. 

**Table 3:** Composition of membrane substrate.

Sample	PES (wt %)	Zn_2_GeO_4_ (wt %)	NMP (wt %)
**Control**	15.00	0.00	85.00
**Zn_2_GeO_4_/PES**	14.99	0.05	84.96

**Figure 7:**
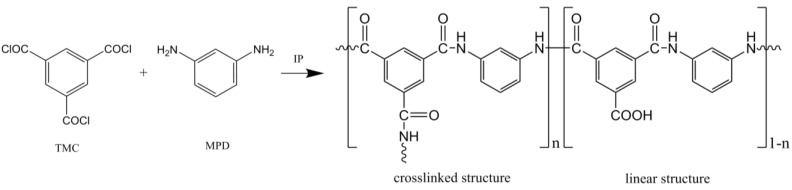
Interfacial polymerization reaction to form polyamide dense layer based on 1,3,5-benzenetricarbonyl trichloride (TMC) and 1,3-phenylenediamine (MPD).

**Interfacial polymerization of TFC Membrane.** The polyamide TFC-FO membranes were fabricated on the top surface of the membrane substrates via interfacial polymerization reaction between MPD and TMC, as shown in Figure 7. The PES membrane was taped on a glass plate, with the skin layer facing away from the glass. The membrane was subsequently immersed in an aqueous solution of 2% MPD for 2 min. The membrane was then removed from the solution and the excess MPD solution on the membrane surface was removed by an air knife. Next, the MPD-saturated support membrane was immersed into 0.15% TMC hexane solution for 1 minute, followed by heat curing step at 60 °C for 10 min. The membrane was then kept in DDI water before further testing.

**Membrane characterization.** Membrane surface and cross-sectional images were obtained using field emission scanning electron microscopy (FESEM; Magellan 400 and Nova NanoSEM 450, FEI, Oregon, USA). Membrane samples were prepared by drying at room temperature and sputter coated with a 0.5 nm thickness of Pt (208h sputter coater, Cressington, Watford, UK). For imaging the cross-section, the membrane was fractured in liquid nitrogen to retain the membrane structure. Structure and morphology of Zn_2_GeO_4_ were confirmed using X-ray diffraction (PXRD; Rigaku MiniFlex, CuKa radiation, Tokyo, Japan) and FESEM. Membrane hydrophilicity was evaluated by contact angle measurement using a contact angle goniometer (OCA15, Dataphysics, Filderstadt, Germany). An average of five measurements for the membrane samples was reported. Zn_2_GeO_4_ nanowire was pressed into a pellet using a hydraulic press (MTI, Richmond, USA) before contact angle measurement. Membrane porosity was determined via gravimetric method using the following equation:

(1) $$\varepsilon =\frac{(m_{1}-m_{2})/\rho_{w}}{(m_{1}-m_{2})/\rho_{w}+m_{2}/\rho_{p}}$$

where m_1_ (g) and m_2_ (g) are the weight of wet and dry membrane, respectively, and ρ_w_ (1.00 g·cm^–3^) and ρ_p_ (0.37 g·cm^–3^) are the densities of water and PES. The molecular weight cut-off (MWCO) of the support membrane was characterized by measuring the rejection of PEG (20, 35, 100, and 200 kDa). A total organic carbon analyzer (TOC-LCSH/CSN with auto-sampler ASI-L, Shimadzu, Tokyo, Japan) was used to measure the amount of organic carbon in permeate. The measured feed (C_f_) and permeate (C_p_) concentrations were used to determine the solute rejection rate (%) and the MWCO:

(2) $$R=\left(1-\frac{C_{p}}{C_{f}}\right)\times 100\%$$

**Figure 8:**
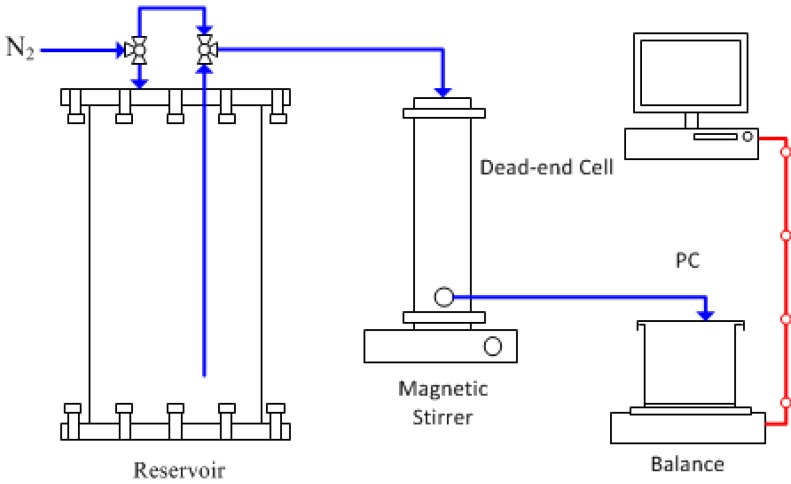
Schematic diagram of bench-scale flux test system.

UF membrane water flux (L·m^–2^·h^–1^ or LMH) was measured using a dead end cell with an effective membrane area of 14 cm^2^ (HP4750 Stirred Cell, Sterlitech, Kent, USA). The bench-scale flux test rig was set-up as reported elsewhere [37]. A schematic diagram of the bench-scale flux test setup is shown in Figure 8. The membrane was precompacted at 150 kPa for at least 30 min before flux test. The water flux test was performed at 100 kPa. 

**Figure 9:**
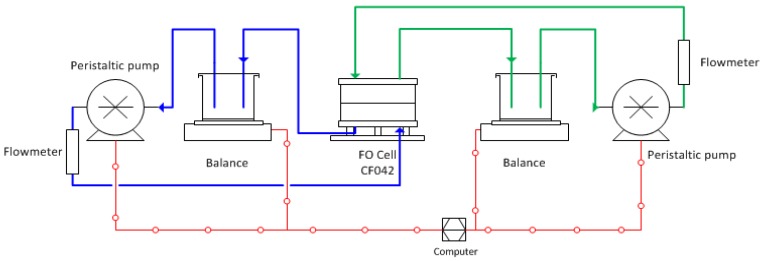
Schematic diagram of bench-scale FO test system.

**Evaluation of FO membrane performance.** The permeability and salt rejection of the FO membrane was determined by testing the membrane in RO mode using the same dead-end cell. The water permeability was obtained by applying transmembrane pressure of 15.51 bar. The salt rejection (R) was determined using 2000 ppm NaCl solution as the feed based on the conductivity measurements of the permeate and feed. The FO performance (water flux and reverse solute flux) of a membrane was evaluated using a bench-scale crossflow permeation FO cell, as shown in Figure 9. The active membrane area in the FO cell (modified Sterlitech CF042 crossflow cell, Kent, USA) is 12.25 cm^2^. Both the FS and DS were circulated at a fixed crossflow rate of 500 mL·min^–1^ on both sides of the membrane. NaCl solution was used as the draw solution, while DDI water was used as the feed. The FO water permeation flux (J_w_; LMH) was determined by measuring the weight changes of the FS with a digital balance (A&D FZ-5000i, Tokyo, Japan) connected to a computer using the following equation:

(3) $$J_{w}=\frac{\Delta m}{\rho A\Delta t}$$

where Δ*m* (g) is the mass of water permeated across the membrane in a predetermined time Δ*t* (h) during the FO process. *ρ* is the density of water and *A* is the effective membrane surface area (m^2^). The reverse solute flux (J_s_; g·m^–2^·h^–1^) was determined from the conductivity measurement of the FS (WTW Cond 730 with conductivity probe LR 325/01, Weilheim Germany) using the following equation:

(4) $$J_{s}=\;\frac{\Delta (C_{t}V_{t})}{A\Delta t}$$

where *C*_t_ (g·L^–1^) and *V*_t_ (L) are the salt concentration and the volume of the feed, respectively. Effects of FS and DS on FO membrane performance were conducted by using different DS concentration (0.5 M, 1.0 M, and 2.0 M) for active-layer-facing-FS (AL-FS) orientations. 

The water flux in FO processes can be modeled by the following equation for FO mode (selective layer against the feed solution) [8]:

(5) $$J_{w}=\frac{1}{K_{m}}\ln\frac{A\pi_{D,b}+B}{A\pi_{F,m}+J_{w}+B}$$

where *π_D,b_* is the osmotic pressures in the respective bulk draw solution, *π_F,m_* refers the feed solution after considering the external concentration polarization effect (ECP) and *K*_m_ is a term signifying the solute resistance to diffusion within the membrane support layer, defined as:

(6) $$K_{m}=\frac{S}{D_{s}}=\frac{t\tau }{D_{s}\varepsilon }$$

where *S* is the structural parameter, *D*_s_ is diffusion coefficient of the solute, and *t*, *τ* and *ɛ* are the thickness, tortuosity, and porosity of the support layer, respectively. *K*_m_ is a measure of the ease of diffusion of a solute into and out of the support layer and thus a measure of the severity of ICP.

## 4. Conclusions

The TFC FO membrane using Zn_2_GeO_4_-nanowire-modified PES membrane as the substrate showed improved performance in RO and FO mode. In RO mode, the water permeability of the membrane was increased by ~45% while retaining the salt rejection. In FO mode, the incorporation of Zn_2_GeO_4_ nanowires to the membrane produced higher J_w_/J_s_ ratio which may be due to the improved interfacial polymerization of polyamide. However, incorporation of Zn_2_GeO_4_ nanowires did not reduce the ICP effects despite considerable improvement over UF flux. This work demonstrates that the incorporation of nanomaterials to the membrane substrate may be an alternative approach to improve the formation of polyamide skin layer to achieve better FO performance. Nonetheless, selection of the nanomaterials is crucial to ensure the modification of the substrate leads to improvement to both the structural parameter of the membrane as well as to the formation of polyamide layer to achieve the best performance. The membrane may also be used as nanofiltration (NF) or RO membrane since they are less affected by ICP effects.
